# Sensors: New Challenges in Spain

**DOI:** 10.3390/s100505028

**Published:** 2010-05-19

**Authors:** Gonzalo Pajares

**Affiliations:** Department of Software Engineering and Artificial Intelligence, Facultad de Informática, Universidad Complutense, 28040 Madrid, Spain; E-Mail: pajares@fdi.ucm.es; Tel.: +34-913947546; Fax: +34-913947547

The main goal of this special issue was to explore sensor technology and its applications in Spain. It is well-known that a reciprocal interrelation exists between sensor technology and the demand for solutions to different problems. Indeed, when a new sensor is developed, it offers a solution to a problem, but also if a problem requires a solution perhaps new sensors or technologies based on existing sensors could be developed.

In this special issue, three main research lines can be considered. The first one includes the sensors’ capabilities, materials and technologies, with special emphasis on the design of sensors based on new materials. The second focuses on the use of sensors for solving problems and also the applications using them. Finally, the third includes several methods and procedures developed for processing the data supplied by sensors, oriented towards the applications. These lines may appear to overlap in some papers. This is because the sequence of operations, normally, closes a cycle. Thus, a sensor device or a network of sensors is used for solving some problem. Then, when trying to solve the problem, new improvements or services might be needed. Based on the above three lines, in what follows, a brief description of the 44 papers published in this special issue is provided, focusing on the most relevant aspects of the increasing and emerging sensor-based technologies, materials, methods and applications in Spain as part of the worldwide research and developments in the field, where Spanish researchers take part actively and play a prominent role.

## Sensor Device Capabilities, Materials and Technologies

*Magnetic* materials offer new application possibilities in civil engineering, biology or space science; *magneto resistances* (including giant ones) and new technologies for developing *magnetometers* (fluxgate) have been also considered. *Ultrasonic*-based and *acoustic* sensors allow recovery of three-dimensional structures and target locations for different purposes. *Sensor networks* and new topologies are well suited for communications, even in underwater environments. In this field, wireless sensor networks for efficiency and security also fall within the new challenges. *Optical* waveguides and fibers based on CMOS and polished polymers have been designed. Distances from an optical camera to a target illuminated with an *infrared* diode allows us to measure distances with high precision. *Sonar* sensors are applied to mobile robot localization. *Multisensor* technologies combining several sensors improve the performance of mono-sensors, as expected; the control of *robots manipulators* under the integration of visual, force/torques and tactile sensors becomes more effective than if they are used separately. Under the multisensory approach, the instrumentation and its design for ocean monitoring based on the physics of the radiometric L-band and the reflectometric one of the Global Navigation Satellite signals has been considered. Specific *electronic* devices for distance and cable length measurements have been designed. Specialized *hardware* implementation for recovering the wave-front phase falls within the scope of this area of research. An overview of the fabrication processes and analytical performance of *ultra-microelectrode arrays* (UMEAs), their characterization and applications, carried out by the Spanish scientific community, are reported. *Bio-chemical* sensors, based on optical, electrochemical, piezoelectric or electro-mechanical devices are appropriately covered. *Optical stereoscopic* sensors are used for measurements in forest environments. A specialized FPGA-based *hardware implementation* has been designed for computing distances to light sources with extremely large telescopes.

## Applications and Problems Addressed

In wireless sensor networks the *security* problem for transmissions is addressed under the design of different strategies; methods to structure their topologies including parallel architectures have also been proposed. *Adaptive topology reorganization* approaches allow maintaining connectivity in underwater wireless sensor networks. A multi-sensor device for *fire detection* in forest and rural environments is built as a node in a wireless sensor network. *Transmission* problems in wireless sensor networks have been studied. A distributed sensor network for the control of a *bioclimatic* house is also considered. The *identification of molecules* in biochemical applications has been also addressed using optical sensors. Laser sensors are proposed as range sensors for solving the *robot navigation* problem, for precise *calibration* in an articulated arm coordinate measuring machine and to *localize the position* of a drill tool. Image sensors can be *calibrated* based on incoherent optical fiber bundles. *Sensor fusion* for designing adaptable network *robot* architecture for cooperation has been considered. Simultaneous Localization and Mapping (SLAM) procedures for autonomous *robot* applications belong to another emerging area which has been studied. *Detection* of liquids in flammable atmospheres is possible. *Industrial inspection* for surface defect detection has been also addressed. The multispectral *Advanced Very High Resolution Radiometer* sensor has been applied for computing vegetation index trends in the Iberian Peninsula. Image-based applications have been used for ground *forest inventories* through the Advanced Spaceborne Thermal Emission and Reflection Radiometer (ASTER) and optical stereoscopic devices. Sound time arrival measurements are used for *gunshot detection* in natural Parks. *Urine output* of a critical patient can be automatically supervised and recorded thanks to the design of an appropriate device. Feasibility studies for transmitting data from remote sensors located in the Spanish Antarctic station Juan Carlos I back to Spain have been undertaken. The use of ventilators via oral is a suitable sensor as a non-invasive technique for monitoring respiratory variables.

## Sensor Domain-Oriented Devices and Methods Used for Processing the Data Sensed

*Neuro-fuzzy* is a technique used for recovering 3D environmental information based on ultrasonic sensors. *Points and curves* are extracted as dominant features for robotic navigation purposes with 2D laser range sensors. In sensor networks and networked control systems an *event-based control* strategy becomes suitable. *Photogrammetric* techniques are applied for precise measurements on the decks in recreational ships, saving important production costs. *Conoscopy holography* based on interferometry is proposed as a new technique for precise measurements. *Image processing* techniques are used for coin detection, head tracking and also for forest inventories, the latter being accomplished through use of a stereovision sensor. *Adaptive regression splines* are used for classifying multi-spectral satellite images. *Fault detection and identification* techniques for Unmanned Aerial Vehicles in collaborative contexts are satisfactorily explored. Based on acoustic emissions and applying *least-square techniques*, relative locations among mobile sensors are determined. *Distributed agents* combined with unsupervised learning algorithms are proposed as a solution to solve the node vulnerability problem in sensor networks. Intelligent agent-based architectures are suitable for multisensory integration and communication under a collaborative context. Methods for *geometric corrections* and a new *data format* for quality and storage respectively have been designed for hyperspectral images.

